# Induction of somatic cell haploidy by premature cell division

**DOI:** 10.1126/sciadv.adk9001

**Published:** 2024-03-08

**Authors:** Aleksei Mikhalchenko, Nuria Marti Gutierrez, Daniel Frana, Zahra Safaei, Crystal Van Dyken, Ying Li, Hong Ma, Amy Koski, Dan Liang, Sang-Goo Lee, Paula Amato, Shoukhrat Mitalipov

**Affiliations:** ^1^ Center for Embryonic Cell and Gene Therapy, Oregon Health & Science University, Portland, OR, USA.; ^2^ Department of Obstetrics and Gynecology, the First Affiliated Hospital of Anhui Medical University, No 218 Jixi Road, Hefei, 230022 Anhui, China.; ^3^ Division of Reproductive Endocrinology and Infertility, Department of Obstetrics and Gynecology, Oregon Health & Science University, Portland, OR, USA.

## Abstract

Canonical mitotic and meiotic cell divisions commence with replicated chromosomes consisting of two sister chromatids. Here, we developed and explored a model of premature cell division, where nonreplicated, G_0_/G_1_-stage somatic cell nuclei are transplanted to the metaphase cytoplasm of mouse oocytes. Subsequent cell division generates daughter cells with reduced ploidy. Unexpectedly, genome sequencing analysis revealed proper segregation of homologous chromosomes, resulting in complete haploid genomes. We observed a high occurrence of somatic genome haploidization in nuclei from inbred genetic backgrounds but not in hybrids, emphasizing the importance of sequence homology between homologs. These findings suggest that premature cell division relies on mechanisms similar to meiosis I, where genome haploidization is facilitated by homologous chromosome interactions, recognition, and pairing. Unlike meiosis, no evidence of recombination between somatic cell homologs was detected. Our study offers an alternative in vitro gametogenesis approach by directly reprogramming diploid somatic cells into haploid oocytes.

## Teaser

Creating haploid eggs via somatic cell nuclear transfer is an alternative path to in vitro gametogenesis.

## INTRODUCTION

Chromosomes serve as fundamental units for organizing genomes and are key vehicles for even replication and distribution of genetic information during cell divisions. In somatic cells, the diploid genome, containing two sets of parental chromosomes, is maintained through the duplication and subsequent segregation of sister chromatids during mitotic cell cycles. This process requires two crucial events: the establishment of cohesion between sister chromatids during DNA replication (*1*–*4*) and the presence of a bipolar spindle apparatus that effectively separates each sister chromatid to opposite poles during cell division. Chromosome duplication and segregation during mitotic cell cycles are interconnected processes, as one cannot occur without the other (*5*). The exception to that is the first meiotic division, a unique reductional cell division that segregates whole homologous chromosomes, each consisting of two sister chromatids, to daughter cells. The second meiotic division follows promptly after the first division without intermediate S phase and resembles an equational division pattern of mitosis, where sister chromatids segregate.

Current efforts to generate functional haploid gametes from diploid somatic cells, termed in vitro gametogenesis (IVG), depend on induction of meiosis in vitro (*6*–*8*). Our recent work introduced an alternative IVG approach using haploidization during somatic cell nuclear transfer (SCNT) (*9*). We modified the conventional SCNT method, using metaphase activity in enucleated MII oocytes to induce so-called premature cell division, resulting in subsequent ploidy reduction in transplanted G_0_/G_1_ diploid somatic cell genomes by bypassing the S phase. Fertilization of metaphase-arrested SCNT-oocytes with sperm triggers somatic chromosome segregation into a pseudo polar body 2 (PB2) and a female pronucleus, while the sperm genome forms a male pronucleus. We demonstrated that such mouse zygotes, containing somatic and sperm pronuclei, may develop into diploid blastocysts, embryonic stem cells, and live offspring, albeit at very low efficacy (*9*). While our previous study established the proof of principle that haploidy in somatic cell genomes can be induced experimentally by premature cell division, the mechanisms and factors influencing spindle organization and chromosome segregation in these unique settings remain unclear.

In contrast to the metaphase spindle-chromosomal complexes in canonical meiotic I and II and mitotic cell divisions, where each chromosome consists of two sister chromatids, here, we investigate chromosome segregation dynamics after SCNT-mediated induction of premature metaphase spindles from the G_0_/G_1_ chromatin of somatic cells, devoid of sister chromatids and replication-mediated cohesion. Through a paired analysis of both daughter cells and accurate sequencing of homologous chromosomes from diverse genetic strains, our study provides insights into the intrinsic potential for homolog recognition and segregation in the absence of meiosis I machinery. Moreover, our approach offers a valuable platform for future research on reconstructing somatic chromosomes during SCNT-mediated IVG.

## RESULTS

### Residual metaphase activity in enucleated MII oocytes facilitates spindle formation and sister chromatid segregation in transplanted nuclei

Mature mammalian oocytes remain arrested at the metaphase of second meiotic division (MII) until sperm entry during fertilization, which triggers release from the MII arrest and segregation of sister chromatids into the PB2 and the zygote. We have previously developed approaches for removal (enucleation) of chromosomes and spindles in MII-arrested oocytes while retaining the residual M-phase activity in the cytoplasm (MII cytoplast) in mouse and primate species (*10*–*13*). In humans, transplantation of donor MII spindles into cytoplasts allows integration of spindles and accurate sister chromatid segregation upon in vitro fertilization. Moreover, we showed that MII cytoplasts could induce de novo formation of functional spindles from transplanted DNA of polar body 1 (PB1) (*14*). In humans, use of donor MII cytoplasts as a machinery for accurate chromosome segregation has been clinically applied to replace mutant mitochondrial DNA or deficient cytoplasm in patient oocytes, resulting in the birth of healthy children (*15*).

**Fig. 1.**
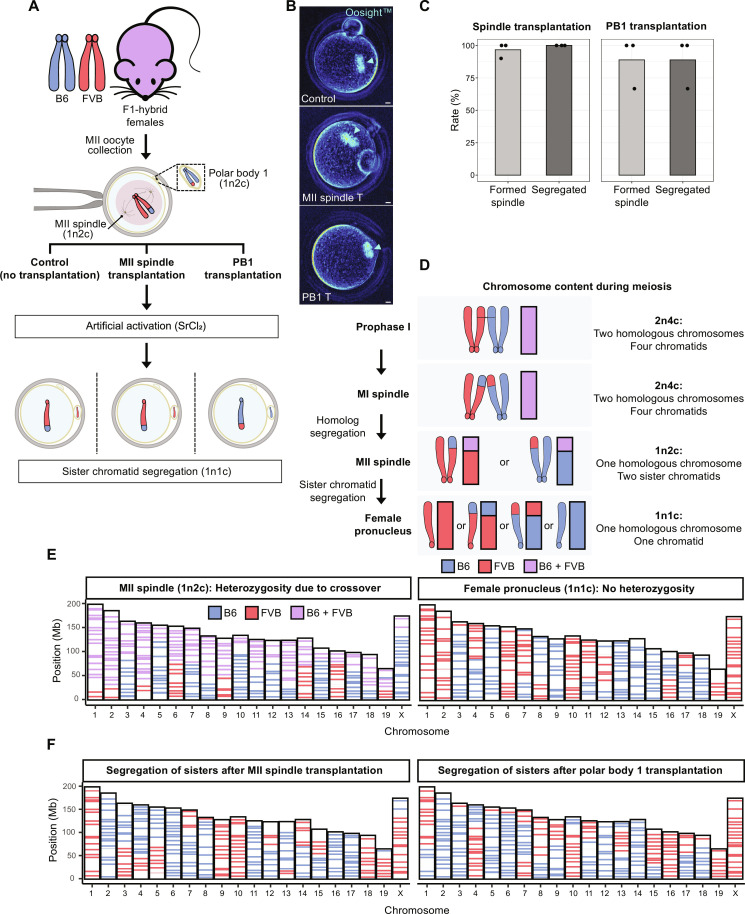
MII cytoplasts support partitioning of transplanted 1n2c genomes by segregation of sister chromatids. (**A**) Schematic of experiments investigating segregation of transplanted 1n2c genomes. (**B**) Spindle formation visualized using noninvasive Oosight live imaging. Control MII oocyte (top) with typical MII spindle pointed by arrowhead. Transplanted MII spindles (middle) or PB1 (bottom) formed typical spindles 2 hours after transplantation. Scale bar, 10 μm. (**C**) Spindle formation and genome segregation rates after MII spindle and PB1 transfer. Bar plots show the mean of three independent experiments (total *n* = 23 for MII spindle transplantation; total *n* = 7 for PB1 transplantation). (**D**) Illustration of chromosome ploidy (n) and chromatid number (c) at various stages of meiosis. (**E**) Analysis of chromosome origin and segregation in control, intact MII oocytes, and zygotes from B6/FVB F1 hybrid females via single-cell AmpliSeq sequencing. Left: Sequencing of DNA from MII spindles consisting of single set of chromosomes with two sister chromatids (1n2c). Right: Typical AmpliSeq profile for a haploid female pronucleus (1n1c) in zygotes after artificial activation of hybrid MII oocytes. (**F**) Analysis of chromosome segregation in zygotes after MII or PB1 transplantation. Left: Female pronucleus after transplantation of MII spindles. Right: Female pronucleus after transplantation of PB1. Note alternating homozygous B6 and FVB regions in individual chromosomes specific to 1n1c genomes.

Here, we isolated mouse MII spindles or PB1 from hybrid B6/FVB (F1, C57BL-6/J, and FVB/NJ) oocytes, representing 1n2c ploidy, and transplanted into MII cytoplasts derived from BDF1 females (Fig. 1A). Live imaging of reconstructed oocytes demonstrated formation of visible spindles within 2 hours in 95.6% (22 of 23) oocytes with transplanted spindles and 85.7% (6 of 7) oocytes with PB1 (Fig. 1A and table S1). Upon artificial activation, 100% (22 of 22) of transplanted MII spindles and 88.9% (5 of 6) of PB1 extruded the PB2 and formed the female pronucleus, indicating that MII cytoplasts retain capacity for asymmetric cytokinesis (Fig. 1C and table S1).

We then investigated whether chromosome segregation of transplanted 1n2c genomes results in the typical meiotic II segregation of sister chromatids by individually sequencing each mouse chromosome in the PB2 and the pronucleus of zygote. To differentiate chromosome origin, we performed whole-genome sequencing of B6 and FVB mice and generated a consensus catalog of chromosome-specific variants (fig. S1, A and B). On the basis of this information, we developed a cost-effective, custom sequencing assay (AmpliSeq), that enabled targeted sequencing of multiple regions on each chromosome, with each region covering, on average, 9 variants (min, 1; max, 21) (fig. S1C). Applying this AmpliSeq assay on bulk DNA controls revealed consistent targeting and capture of 74% originally designed regions (281 of 381). When applied to control DNA isolated from a single fibroblast or a cumulus cell, a somatic cell surrounding oocytes, results revealed that despite occasional allelic dropouts during whole-genome amplification (WGA) (*16*), sequencing of multiple regions per chromosome compensated the fractional loss of some regions allowing to correctly infer chromosome identity (fig. S1D). In addition, the AmpliSeq assay could detect large chromosomal crossover events typical of gametes.

As expected, sequencing of 1n2c genomes of MII spindles from the B6/FVB F1-hybrid oocytes revealed regions of homozygosity and heterozygosity as a result of crossover recombination during meiosis I (Fig. 1, D and E). Parthenogenetic activation of intact control MII oocytes produced zygotes with a female pronucleus and a PB2, each containing segregated sister chromatid genomes (1n1c). Sequencing of DNA from the pronucleus revealed that all 20 chromosomes now appear homozygous, but some individual chromosomes consisted of interchanging B6 and FVB regions (Fig. 1E). Similar to intact controls, sequencing of zygotic pronuclei after division of transplanted MII spindles or PB1 demonstrated normal partitioning of 1n2c genomes through faithful segregation of sister chromatids (Fig. 1F).

### Transplantation of G_0_/G_1_ somatic cell nuclei leads to premature spindle formation and chromosome segregation

**Fig. 2.**
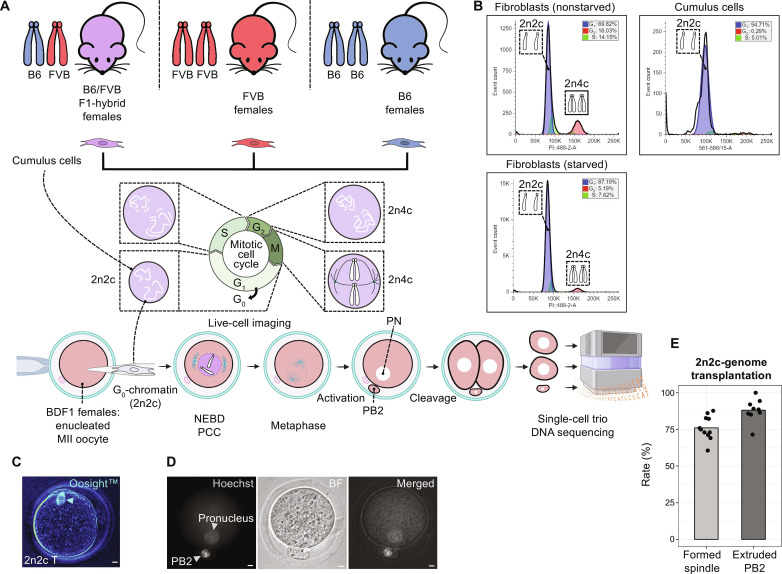
Transplantation of nonreplicated, diploid 2n2c genomes into MII cytoplasts leads to rapid onset of premature spindles followed by chromosome segregation. (**A**) Schematic of experiments interrogating premature cell division and chromosome segregation in diploid but nonreplicated (2n2c) somatic cell genomes. (**B**) Cell cycle analysis by flow cytometry of somatic cell nuclei labeled with propidium iodide. Majority of cumulus cells (right); or fibroblasts after serum starvation (lower left) were at the G_0_/G_1_ phase. (**C**) De novo metaphase spindle formation observed by live Oosight imaging 2 hours after SCNT. Scale bar, 10 μm. (**D**) Partitioning of transplanted 2n2c somatic genomes into a PB2 and a pronucleus after activation of SCNT oocytes. Left: DNA signal (Hoechst 33342). Middle: Bright-field (BF) image. Right: Merged images. Scale bar, 10 μm. (**E**) Rates of de novo premature metaphase spindle induction in SCNT oocytes followed by the PB2 extrusion and pronucleus formation. Bar plots show mean values derived from nine independent SCNT experiments with G_0_/G_1_ cumulus cells (mean of 70 SCNT oocytes per experiment, total 626 SCNT oocytes).

Next, we investigated whether transplantation of diploid but nonreplicated somatic cell genomes composed of single chromatids (2n2c) into MII cytoplasts can trigger premature metaphase spindle formation and subsequent chromosome segregation (Fig. 2A). We used cumulus cells isolated from MII oocytes of B6/FVB hybrid females as 2n2c donor genomes for SCNT (*17*–*19*). Flow cytometry analysis of DNA content in cultured dermal fibroblasts showed that 70% of cells were in the G_0_/G_1_, 14% were in the S, and 16% were in the G_2_ phase of the cell cycle (Fig. 2B). Upon serum starvation, proportion of cells in the G_0_/G_1_ phase increased to 87%. More than 90% of cumulus cells isolated from mature MII oocytes also appeared at the G_0_/G_1_ phase of the cell cycle (Fig. 2C). Using a modified mouse SCNT approach described by us earlier (*9*), we generated SCNT oocytes with cumulus cell nuclei and examined de novo spindle formation and chromosome partitioning after artificial activation. On average, 77% of SCNT oocytes exhibited nuclear envelope breakdown, rapid chromosome condensation, and de novo formation of bipolar spindle-chromosome complexes (Fig. 2C and table S1). Subsequent artificial activation of SCNT oocytes with visible spindles led to the extrusion of a PB2 and formation of a single pronucleus in 88% of zygotes (Fig. 2, D and E). This outcome was similar to PB1 transplantations, demonstrating the capability of MII cytoplasts to induce de novo formation of premature metaphase spindles and subsequent segregation of chromosomes from nonduplicated 2n2c somatic cell genomes.

**Fig. 3.**
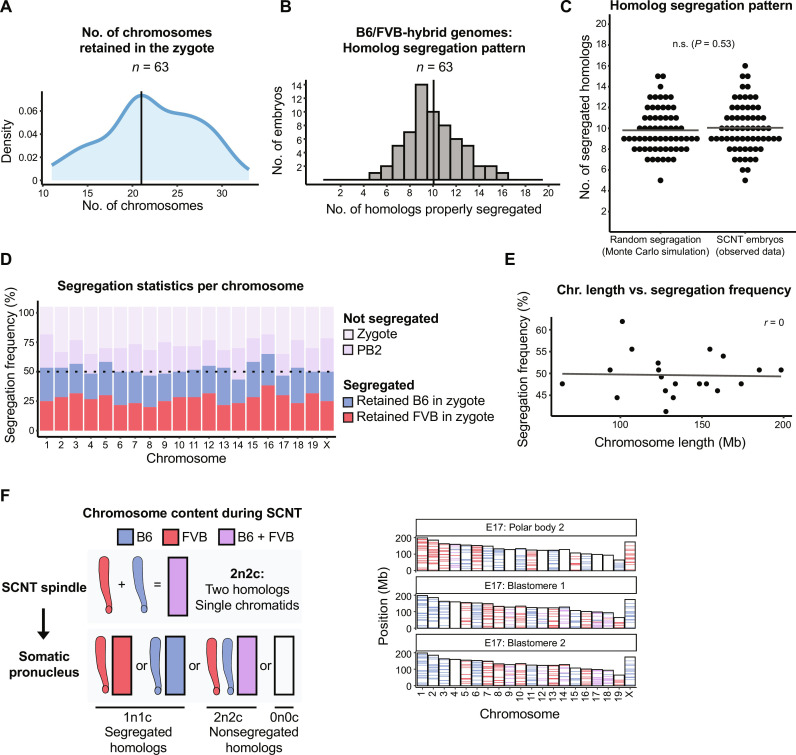
Random chromosome segregation after premature division of diploid, nonreplicated 2n2c hybrid genomes. (**A**) Number of chromosomes retained within a zygote upon partitioning of diploid, nonreplicated (2n2c) genomes in SCNT oocytes generated from hybrid B6/FVB somatic cell nuclei. Vertical line represents the mode number (21) derived from analysis of 63 SCNT embryos collected from eight independent experiments. (**B**) Number of homologous chromosomes properly segregated in 63 individual SCNT embryos. Vertical line indicates the mean number (10) of properly segregated homologs. (**C**) Comparison of homolog segregation patterns observed in SCNT embryos with random distribution expected from the Monte Carlo simulation. Horizontal lines represent the mean; n.s., not significant (*P* = 0.53, Wilcoxon rank sum test). (**D**) Segregation and nonsegregation statistics in SCNT embryos per individual mouse homolog. Horizontal dash line illustrates a hypothetical 50% chance for a pair of homologs to segregate (random segregation). (**E**) No linear relationship seen between chromosome length and homolog segregation frequency; *r*, Pearson correlation coefficient. (**F**) Illustration of the chromosome content (left) and segregation pattern in the SCNT embryo no. 17 (right) assessed by AmpliSeq analysis of DNA isolated from the PB2 and both blastomeres (trio). Only SCNT embryos producing sequence reads for all chromosomes in all trio samples (PB2 and two sister blastomeres) were included in analyses. Typical B6 or FVB sequence across all chromosomal regions in SCNT samples indicates absence of crossover recombination.

We then asked how diploid somatic chromosomes composed of a single chromatid (2n2c) in SCNT oocyte spindles segregate during the cell division. SCNT oocytes reconstructed with B6/FVB-hybrid cumulus cells were activated allowing cytokinesis and cultured to the two-cell stage embryos. We individually collected both sister blastomeres and a PB2 in each SCNT embryo and processed for AmpliSeq (Fig. 2A). To ensure data integrity and reliability, only embryos that produced sequencing reads for all 40 individual chromosomes in all three samples (trio: two blastomeres and the PB2) were included in the final analysis of chromosome segregation. Of the 122 SCNT embryos collected, 59 samples were excluded because of PB2 DNA degradation or artifacts of WGA and sequencing library preparation. Analysis of 63 SCNT embryos, which provided a full set of trio sequencing for all 40 chromosomes, revealed that premature cell divisions retained roughly half (median *n* = 21) of chromosomes within a zygote (in blastomeres), while extruding 19 chromosomes into a PB2 (Fig. 3A and table S1). These findings demonstrate that premature segregation of chromosomes from 2n2c somatic cell genomes results in ploidy reduction roughly in half, with a 5% error rate.

### Hybrid genomes undergo random chromosome segregation following premature cell division

We further analyzed the frequency and accuracy of homologous chromosome segregation during premature partitioning of 2n2c chromosomes from hybrid cumulus nuclei. On average, only 10 of total 20 mouse somatic homologous chromosome pairs were properly segregated into a zygote and a PB2 (Fig. 3B and table S1). The maximum number of properly segregated homologs seen in 63 SCNT embryos was 16, while the minimum was 5. To assess whether the observed somatic homolog segregation patterns in SCNT embryos could be the result of random events, we performed a Monte Carlo simulation test for random (by chance) segregation of 20 homolog pairs in 63 samples. This simulation revealed that, on average, only 10 homolog pairs would be expected to properly segregate to daughter cells by chance, with individual cases ranging from 6 to 16 homologous chromosomes (Fig. 3C). Comparison of this random distribution to that observed in our SCNT embryos did not reveal any statistically significant differences, indicating that segregation of 2n2c chromosomes in premature spindles composed of hybrid B6/FVB genomes occurred randomly (Fig. 3C). Evaluation of homolog pairs that did not segregate suggested a slight propensity for a zygote (blastomeres) to retain both homologs compared to extrusion of both homologs to the PB2 (Fig. 3D). We also evaluated segregation patterns for individual chromosomes and found no correlation between the chromosome length and homolog segregation frequency (Fig. 3E and table S1). Separate assessment of maternal (B6) and paternal (FVB) chromosomes segregation indicated random assortment of parental homologs to the PB2 and the zygote. Chromosome composition analysis among segregated homologs indicated that all chromosomes retained original B6 or FVB patterns, indicating the lack of crossover recombination (Fig. 3F). These findings indicate that despite the overall somatic chromosome ploidy reduction induced by premature cell division of 2n2c genomes, the partitioning of nonreplicated homologous chromosomes from B6/FVB hybrids occurred randomly.

### Genetically identical homologs facilitate accurate segregation and haploidization

**Fig. 4.**
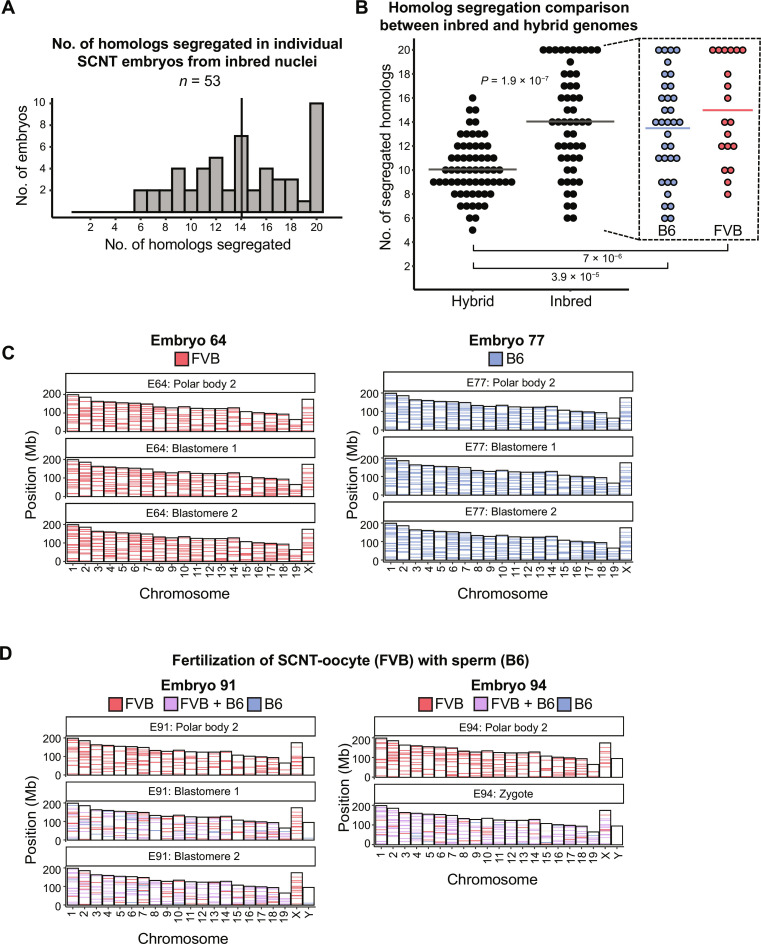
Partitioning of diploid, nonreplicated 2n2c chromosomes from inbred genetic background results in haploid chromosome composition in daughter cells. (**A**) Number of homologous chromosomes properly segregated in individual SCNT embryos (*n* = 53) composed of inbred FVB or B6 genomes. Vertical line indicates the mean (14). (**B**) Comparison of homologous chromosome segregation patterns between SCNT embryos composed of inbred (*n* = 53) versus hybrid (*n* = 63) somatic cell nuclei. Horizontal lines represent the mean; *P* = 1.85 × 10^−7^. Statistical significance was assessed by nonparametric, Wilcoxon rank-sum test. (**C**) Representative AmpliSeq chromosome sequencing data generated for the SCNT embryo no. 64 (left) and no. 77 (right) generated from FVB and B6 nuclei, respectively. (**D**) AmpliSeq chromosome sequencing profiles for two fertilized SCNT embryos showing proper segregation of all 20 homologs.

It is possible that the genome sequence divergence between B6 and FVB mouse strains negatively affected the pairing and subsequent faithful segregation of homologous chromosomes in hybrids. We reasoned that having homologs with high sequence homology could support better segregation outcomes. Because in parental inbred mouse strains, homologous chromosomes are nearly identical to each other, we tested our assumption by repeating SCNT experiments with cumulus cells recovered from B6 or FVB mice. Chromosome segregation analysis was performed on PB2 and either zygote or two sister blastomere DNA from 53 SCNT embryos (34 B6 and 19 FVB) that met the inclusion criteria described earlier. The results revealed that the mean number of properly segregated homologous chromosomes (14) from inbred genetic backgrounds was significantly higher (Wilcoxon rank sum test, *P* = 1.85 × 10^−7^) than that observed for hybrid genomes or the expected random distribution patterns (Fig. 4, A and B, and table S1). Complete haploidization of somatic cell genomes through proper segregation of all 20 mouse homolog pairs was observed in 19% (10 of 53) of SCNT embryos (Fig. 4C). These results suggest that premature segregation of diploid but nonreplicated 2n2c genomes composed of nearly identical homologs is not random but rather resembles ploidy reduction expected in typical meiosis I. Furthermore, these observations suggest that premature spindles in SCNT oocytes may support sequence-driven recognition and pairing of homologs, facilitating faithful haploidization.

We further expanded our investigation to determine whether normal diploidy could be restored in haploid SCNT zygotes fertilized with sperm. SCNT oocytes generated from FVB cumulus cells were fertilized with sperm from B6 males, resulting in efficient PB2 extrusion (77%) and formation of both somatic and male pronuclei in SCNT zygotes. Chromosome analysis of 14 fertilized SCNT embryos revealed that 2 (14%) embryos extruded all 20 FVB chromosomes into the PB2, while sister blastomeres exhibited B6/FVB heterozygosity for all chromosomes (Fig. 4D). Remaining SCNT embryos showed partial segregation of somatic homologs (table S1). These results suggest that fertilization of SCNT oocytes with sperm can simultaneously induce activation and segregation of homologs and restore normal diploidy in zygotes consisting of somatic and sperm homologs.

## DISCUSSION

Our study reveals the feasibility of inducing partitioning of diploid but nonreplicated somatic cell genomes in SCNT platform. The G_0_/G_1_ nuclei introduced into MII cytoplasts undergo rapid remodeling, including nuclear envelope breakdown, premature chromatin condensation, and spindle formation (*20*). Because conventional SCNT protocols inhibit PB2 segregation, it remained unclear whether premature spindles were functional and capable of inducing somatic genome partitioning. We recently demonstrated that fertilization of SCNT oocytes can produce diploid embryos and live mouse offspring consisting of somatic and sperm genomes (*9*). In contrast to naturally occurring mitotic or meiotic spindles, the chromosomes in SCNT oocytes are not replicated (2n2c), which raises questions about how these single-chromatid chromosomes align and attach to microtubules. Through comprehensive sequencing analysis of all 40 mouse chromosomes, we observed that most of the SCNT spindles induce partitioning of chromosomes into the PB2 and the pronucleus leading to the ploidy reduction. However, the segregation pattern of somatic chromosomes in B6/FVB hybrids appeared to be random. Unexpectedly, the partitioning of homologous chromosomes in SCNT oocytes composed of genetically identical homologs from inbred strains showed a higher degree of fidelity, suggesting the existence of homology-recognizing machinery that regulates chromosome associations, pairing, and segregation.

Meiosis I is the only known natural reductional cell division that ensures proper segregation of homologous chromosomes to produce haploid gametes. Pairing, synapsis, and crossover recombination are the key native events that evolved to facilitate proper homolog segregation by converting sister chromatid cohesion produced during premeiotic S phase into a mechanism that holds paired homologs together (*21*–*25*). Pairing acts as the initial alignment of homologs and sets the stage for synapsis, which connects homologs along their entire length through the formation of synaptonemal complex. This structure preserves homolog proximity necessary for repairing hundreds of double-strand breaks (DSBs) throughout the genome, some of which mature into chiasmata, a physical manifestation of interhomolog recombination. These connections, established by the combined effect of interhomolog crossover and sister chromatid cohesion, prevent paired homologous chromosomes from drifting apart, allowing proper alignment on opposite poles of meiotic spindle until tension on kinetochore-microtubule complexes is formed ensuring homologs biorientation and faithful segregation during anaphase I upon the release of cohesion distal to chiasmata (*5*, *26*, *27*). Failure to establish chiasma between a pair of homologs may result in their random segregation leading to aneuploidy if both homologs are pulled to the same pole of MI spindle (*28*).

Homolog pairing during meiosis I is preceded by programmed DNA DSBs that, in turn, recruit DNA repair and homologous recombination machinery necessary for sensing and pairing on the basis of DNA sequence homology at the end of meiotic prophase (*29*). Therefore, it is believed that DSBs evolved as an efficient mean of recognition and pairing of homologs (*5*, *23*, *27*). Mechanisms involved in homolog recognition in our SCNT model of premature cell division remain unknown, but the lack of recombination suggests the absence of programmed DSBs and thus, a DSB-independent mode of homolog pairing. Previous studies also revealed exceptions from the canonical DSB-dependent pairing mechanisms showing that in some species homolog pairing and synapsis can occur without DSBs. Moreover, although synapsis is required for stabilized pairing along the entire lengths of chromosomes, there may be additional mechanisms taking place that can promote local stabilization of pairing, independent of synapsis (*30*). For instance, in species such as *Caenorhabditis elegans* and *Drosophila*, homologous chromosome pairing, synapsis and/or segregation may occur normally even in the complete absence of DSBs and recombination (*30*–*33*). In *C. elegans* and *Drosophila* female meiosis, pairing and synaptonemal complex formation occur independently of DSBs and recombination, while in *Drosophila* male meiosis, the entire meiotic program occurs without recombination (*34*–*36*). Although the molecular basis for homolog pairing and segregation in these organisms remains not completely understood, studies in *Drosophila* males indicated that DSB-independent processes of achiasmate segregation may rely on pairing of homologs facilitated by repetitive DNA sequences that act as pairing sites and promote interactions between intact nonrecombinant homologous chromosomes (*37*–*41*). Recently, the lack of homology in pericentromeric repetitive DNA was proposed as a reason behind hybrid incompatibility in *Drosophila* (*42*). It was suggested that *Drosophila* females could also exhibit limited ability for achiasmate segregation without detectable physical pairing (*39*). In *C. elegans*, chromosomes enter meiosis unpaired yet quickly undergo DSB-independent pairing facilitated and stabilized by homolog recognition regions (*30*, *43*). Furthermore, inactivation of meiotic cohesion does not prevent initial alignment of homologs (*44*). Even in the absence of DSBs-mediated pairing (*C. elegans* and *Drosophila* female) and crossover recombination (*Drosophila* males), the outcome of genome haploidization in these cases is indistinguishable from what is observed in canonical DSB-dependent homolog segregation. Mammalian meiosis is believed to follow a strictly DSB-dependent pairing, recombination, and segregation of homologs. Our experimental model suggests the presence of alternative mechanisms that might facilitate pairing and segregation of nonreplicated homologous chromosomes and allowing formation of interhomolog associations that can replace chiasmata-mediated segregation. Recent observations in *Rec8* and *Rad21L* (meiosis-specific cohesins) knockout mice also suggest that a substantial portion of mouse oocytes progressed into the pachytene-like stage exhibiting homolog pairing and synapsis despite the absence of cohesion (*45*). Last, the possibility of single chromatid homolog segregation has been reported for reverse meiosis, where sister chromatids segregate at meiosis I, followed by reductive meiosis II that separates nonduplicated homologs. This pattern was described in human oocytes for several individual chromosomes (*46*), suggesting that a similar mechanism may exist in MII cytoplasm supporting the haploidization by premature chromosome segregation in SCNT oocytes.

It should be also noted that haploidy induced by premature cell division is ineffective and sequence divergence between B6 and FVB homologs precluded faithful segregation, while canonical meiosis in B6/FVB hybrid oocytes occurs normally. This suggests that single-chromatid chromosome pairing forced by premature spindle formation during SCNT is insufficient for proper homolog recognition, pairing, and/or maintaining their stable associations in hybrids. Therefore, additional stimuli (e.g. programmed DSBs) will likely be required to enhance recognition of homologous chromosomes and their proper pairing and synapsis in hybrid SCNT oocytes. In addition, lack of recombination between B6 and FVB chromosomes in hybrids observed in our study suggests that native meiotic recombination machinery is absent in MII cytoplasts.

For the success of IVG, somatic cells need to acquire several critical characteristics of oocytes, including the following: (i) epigenetic identity of mature oocytes, (ii) cytoplasmic maternal factors of oocytes critical for induction of totipotency and preimplantation embryo development, (iii) haploidy, (iv) recombination, and (v) imprinting reset. SCNT-mediated oocyte haploidization may offer advantages as it relies on the donor cytoplasm and maternal factors derived from in vivo matured oocytes. The developmental potential of diploid SCNT oocytes/embryos has been well demonstrated by production of live “cloned” offspring in various mammalian species, including nonhuman primates (*17*, *19*, *47*–*49*). In humans, we demonstrated that conventional SCNT allows the generation of histocompatible embryonic stem cells (*11*).

Here, we show that premature metaphase spindles formed in SCNT oocytes are capable of discarding one set of the parental homologs into the PB2 upon cell division. Moreover, SCNT oocytes can be fertilized with sperm, restoring the normal diploid genome in zygotes consisting of somatic and sperm chromosomes. However, the efficacy of this IVG approach remains extremely low, likely because of cumulative errors in homolog segregation, reprogramming, and imprinting (*9*).

### Limitations of the study

Although our data clearly showed higher rates of faithful genome segregation when maternal and paternal homologs had the same genetic origin, further studies are needed to uncover the exact mechanism behind the trend observed. Other experimental approaches, such as one using live-cell imaging, as well as evaluating chromosome segregation patterns in other inbred mouse strains, may provide complementary insights into DSB-independent homolog segregation mechanisms. It is also worth noting that the SCNT approach used here may not accurately reflect the multifactorial process of mammalian meiosis per se, as it involves manipulating cells and chromosomes outside of the natural context. Instead, we believe that our findings reflect the intrinsic potential of highly homologous chromosomes for self-recognition, pairing, and maintaining stable achiasmate associations that result in their faithful segregation.

## MATERIALS AND METHODS

### Animals

All animal experiments were approved by the Institutional Animal Care and Use Committee at Oregon Health & Science University (OHSU). Mice were housed at OHSU under controlled lighting conditions (12-hour light/dark cycles) and specific pathogen–free conditions.

### MII oocytes collection and cumulus cell preparation

Six- to eight-week-old BDF1 (cross between C57BL/6 J female × DBA/2 J male), C57BL-6/J, FVB/NJ, and B6/FVB F1-hybrid (cross between C57BL/6 J female × FVB/NJ male) females were superovulated by injecting with 5 IU of pregnant mare’s serum gonadotropin and human chorionic gonadotropin (hCG). The oviducts were excised 14 hours after hCG injection to collect the cumulus-oocyte complexes, which were then briefly exposed to a medium containing 0.1% hyaluronidase for disaggregation. Dispersed cumulus cells and MII oocytes were separated and maintained in Hepes-Chatot-Ziomek-Bavister (H-CZB) + 0.5% bovine serum albumin and Potassium Simplex Optimized Medium (KSOM) medium (Millipore) respectively until SCNT. MII cytoplasts were prepared using BDF1 oocytes, while the cumulus cells from C57BL-6/J, FVB/NJ, and B6/FVB F1-hybrids served as the source of donor nuclei for SCNT.

### Primary culture of fibroblasts and bulk DNA extraction

Dermal fibroblasts were isolated and cultured from mouse tail or ear punch biopsies. The biopsied tissues were minced into small pieces, followed by enzymatic digestion with collagenase at 37°C for 20 to 30 min. The resulting tissue fragments were rinsed, plated, and cultured in Dulbecco’s modified Eagle’s medium supplemented with 10% fetal bovine serum. Fibroblasts cultures were grown and expanded up to three passages. Genomic DNA was extracted from the cell pellets using a commercial DNA extraction kit (Qiagen no. 158023) following the manufacturer’s manual.

### Somatic cell nuclear transfer

All oocyte/embryo imaging and micromanipulations were conducted on inverted microscopes (Olympus IX71) equipped with stage warmers (Tokai Hit), XyClone laser objectives (Hamilton Thorne), Oosight spindle imaging system (Hamilton Thorne), and Narishige micromanipulators. For oocyte enucleations, cumulus cell–free, mature MII oocytes from B6D2F1 females were placed into a 30-μl droplet of Hepes-CZB medium (*50*) containing cytochalasin B (5 μg/ml) and 1.25 mM caffeine and covered with tissue culture oil on a glass bottom dish. An optical Oosight birefringence system was used for detection of spindle-chromosome structures. An oocyte was positioned using a holding pipette so that the spindle was situated at 2 to 4 o’clock. The zona pellucida next to the spindle was laser drilled, and an enucleation pipette was introduced through the slit. The spindle was extracted by aspiration into the pipette with a minimal amount of cytoplasm, surrounding plasma membrane, and discarded. Dispersed cumulus cells or fibroblasts at G_0_/G_1_ phase of the cell cycle were drawn into a nuclear transfer pipette, briefly exposed to HVJ-E extract (Cosmo Bio USA), and placed deep into MII cytoplasts (enucleated MII oocytes), ensuring that the donor cell is completely surrounded by the oocyte membrane. SCNT couples were incubated for 30 min to allow fusion and evaluated for de novo spindle formation 2 hours later using the Oosight imaging system.

### Oocyte activation

SCNT oocytes were subjected to the modified artificial activation treatment that allows PB2 extrusion. The treatment involved incubating the oocytes in Ca^2+^-free CZB medium containing 10 nM SrCl_2_ and 10 nM trichostatin A (TSA) (Sigma-Aldrich) at 37°C in 5% CO_2_ 6% O_2_, and 89% N_2_ for 4 hours. The activated SCNT oocytes were then transferred to KSOM medium with TSA and cultured for an additional 4 hours. Pronuclear formation and PB2 extrusion were evaluated, and the embryos were further cultured in KSOM medium at 37°C in a gas mixture of 5% CO_2_, 6% O_2_, and 89% N_2_ until the two-cell stage. PB2 and both blastomeres were collected for subsequent analyses.

### Fluorescent analysis of nuclear DNA content

To visualize the extrusion of somatic chromosomes into the PB2, activated SCNT oocytes were stained with the live DNA fluorochrome Hoechst 33342 (Invitrogen) at a concentration of 5 μg/ml. Once the fluorescent dye effectively labeled both the maternal pronucleus and the extruded PB2 (5 to 10 min of incubation), DNA signals were captured and imaged using a fluorescent microscope (Olympus IX70).

Quantitative, flow cytometry–based cell cycle analysis of DNA content was performed on cumulus cells and cycling or serum-starved fibroblasts. Cells were harvested and washed in ice-cold phosphate-buffered saline (PBS) before fixation in ice-cold 66% ethanol. Fixation was performed dropwise while vortexing to ensure proper fixation and minimizing clumping. Fixed cells were stored at +4°C overnight. Next morning, the cells were rehydrated in PBS and treated with ribonuclease I (550 U/ml; Thermo Fisher Scientific) and propidium iodide (550 U/ml; Sigma-Aldrich, P4170) for 30 min in the dark to eliminate RNA signal, leaving only DNA staining with propidium iodide. After incubation, cells were kept on ice until flow cytometry analysis. Flow cytometry analysis was carried out using a BD LSR II. The results were analyzed in Floreada (floreada.io).

### Blastomere isolation and WGA

Following procedures previously described in (*16*), zonae pellucidae of zygotes or two-cell stage embryos were removed by brief exposure to acidic Tyrode solution. Zona-free embryos were exposed to a trypsin solution (0.15% in EDTA-containing Ca- and Mg-free PBS) before manual disaggregation of blastomeres and PB2 using a small-bore pipette. Individual blastomeres and PB2 were transferred into 0.2-ml polymerase chain reaction tubes containing 4 μl of PBS and placed into a freezer at −80°C until further use. WGA was performed using a REPLI-g Single Cell Kit (Qiagen).

### Whole-genome sequencing and AmpliSeq design

For analysis of genomic variants in B6, FVB, and B6/FVB F1-hybrid genomes, the freshly extracted DNA from primary fibroblast cultures was normalized to 500 ng in 25 μl of Resuspension Buffer (RSB) and used for library preparation with Illumina TruSeq DNA Nano kit following manufacturer’s instructions. Paired-end sequencing was performed as 2 × 150 bp at an average autosome coverage depth of 43× on the Illumina NovaSeq 6000. Preprocessing steps using the Genome Analysis Toolkit (*51*) (GATK) were applied to the raw sequencing reads to produce BAM files ready for further analysis. These steps included generating uBAM via FastqToSam (Picard tools v2.26.9; http://broadinstitute.github.io/picard), marking adapters via MarkIlluminaAdapters, converting uBAM to fastq via SamToFastq, mapping to the GRCm38 genome assembly using BWA-MEM (*52*) (v0.7.17), and merging BAM and uBAM with MergeBamAlignment. The aligned reads were then coordinate sorted with SortSamSpark [GATK (*51*) v4.2.6.1], and the duplicate reads were marked with MarkDuplicatesSpark (GATK v4.2.6.1). The resulting BAMs underwent germline variant calling with HaplotypeCaller followed by joint genotyping with GenotypeGVCFs (GATK v4.2.6.1). Genomic variants were also jointly called with FreeBayes (*53*) (v1.3.5) with local left-alignment of indels and further normalized with bcftools (*54*) (v1.14). Additional filters (QUAL > 1 & QUAL/AO > 10 & SAF > 0 & SAR > 0 & RPR > 1 & RPL > 1) were applied to keep only high-confident calls. The resulting sets of variant calling data were used for downstream analysis and characterization of genomic variation in R (v4.0.3; www.r-project.org). Homozygous Ref (B6) and Alt (FVB) calls were compared among three datasets (GATK, Freebayes, and publicly available FVB variants from Mouse Genomes Project, release version 5), to create a consensus catalog of B6 and FVB variants, which served as the basis for the custom AmpliSeq sequencing panel design. Using the defined variant signatures of FVB and B6 chromosomes, a custom AmpliSeq panel targeting 381 genomic regions across all mouse chromosomes was finalized. The selection of targeted loci aimed to cover one genomic region every 7 Mb. The number of targeted loci was normalized by chromosome length, with the exception of chromosome Y. For each 7-Mb interval, a genomic region containing multiple variants within 120 bp but none in the flanking 80 bp (to prevent any variants in the primer binding sites) was selected for inclusion. On average, the targeted loci covered 9 variants per interval (min, 1; max: 21). The average amplicon length was 263 bp, and the total length of all targeted regions was approximately 38 Kb.

### AmpliSeq sequencing and chromosome segregation analysis

For the analysis of chromosome content in collected single-cell blastomeres and polar body samples, the freshly extracted DNA went through WGA using multiple displacement amplification (REPLI-g Single Cell Kit, Qiagen). The amplified DNA concentrations were normalized to 10 ng/μl, and 50 ng was used as an input for library preparation with the AmpliSeq panel and the AmpliSeq Library PLUS kit (Illumina) following the manufacturer’s instructions. Paired-end sequencing was performed on the Illumina MiSeq platform as 2 × 250 bp or on the NextSeq 1000 as 2 × 150 bp to achieve the desired average coverage depth of 250×. Preprocessing steps using GATK were applied to the raw sequencing reads to produce BAM files ready for further analysis. These steps included generating uBAM via FastqToSam (Picard tools v2.26.9), marking adapters via MarkIlluminaAdapters, converting uBAM to fastq via SamToFastq, mapping to the GRCm38 genome assembly using BWA-MEM (v0.7.17), and merging BAM and uBAM with MergeBamAlignment. Duplicate reads were retained. Genomic variants were then found with HaplotypeCaller followed by joint genotyping with GenotypeGVCFs (GATK v4.2.6.1). The resulting set of variant calling data was used for downstream analysis of chromosome segregation in R (v4.0.3). Homozygous loci with variant coverage depth of less than 12× and heterozygous loci with variant coverage depth of less than 24× were considered not amplified and excluded from the inference of chromosome origin.

### Statistical analysis

Statistical analyses were conducted using R (v4.0.3). The homolog segregation rate was compared using the Wilcoxon rank sum test with continuity correction, considering *P* < 0.05 as significant. In addition, the correlation between chromosome segregation frequency and chromosome length was assessed using the Pearson correlation coefficient.
