# Supplementary material for: Induction of somatic cell haploidy by premature cell division
Source: Sci Adv. 2024 Mar 8;10(10):eadk9001. doi: 10.1126/sciadv.adk9001 (PMC10923512; doi:10.1126/sciadv.adk9001)
Supplement: Supplementary file 1 — Fig. S1 Legend for table S1 [file sciadv.adk9001_sm.pdf]

Science Advances
AAAS

Supplementary Materials for

Induction of somatic cell haploidy by premature cell division

Aleksei Mikhalchenko *et al.*

Corresponding author: Shoukhrat Mitalipov, shoukhrat@gmail.com

Sci. Adv. **10**, eadk9001 (2024)
DOI: 10.1126/sciadv.adk9001

The PDF file includes:

Fig. S1
Legend for table S1

Other Supplementary Material for this manuscript includes the following:

Table S1

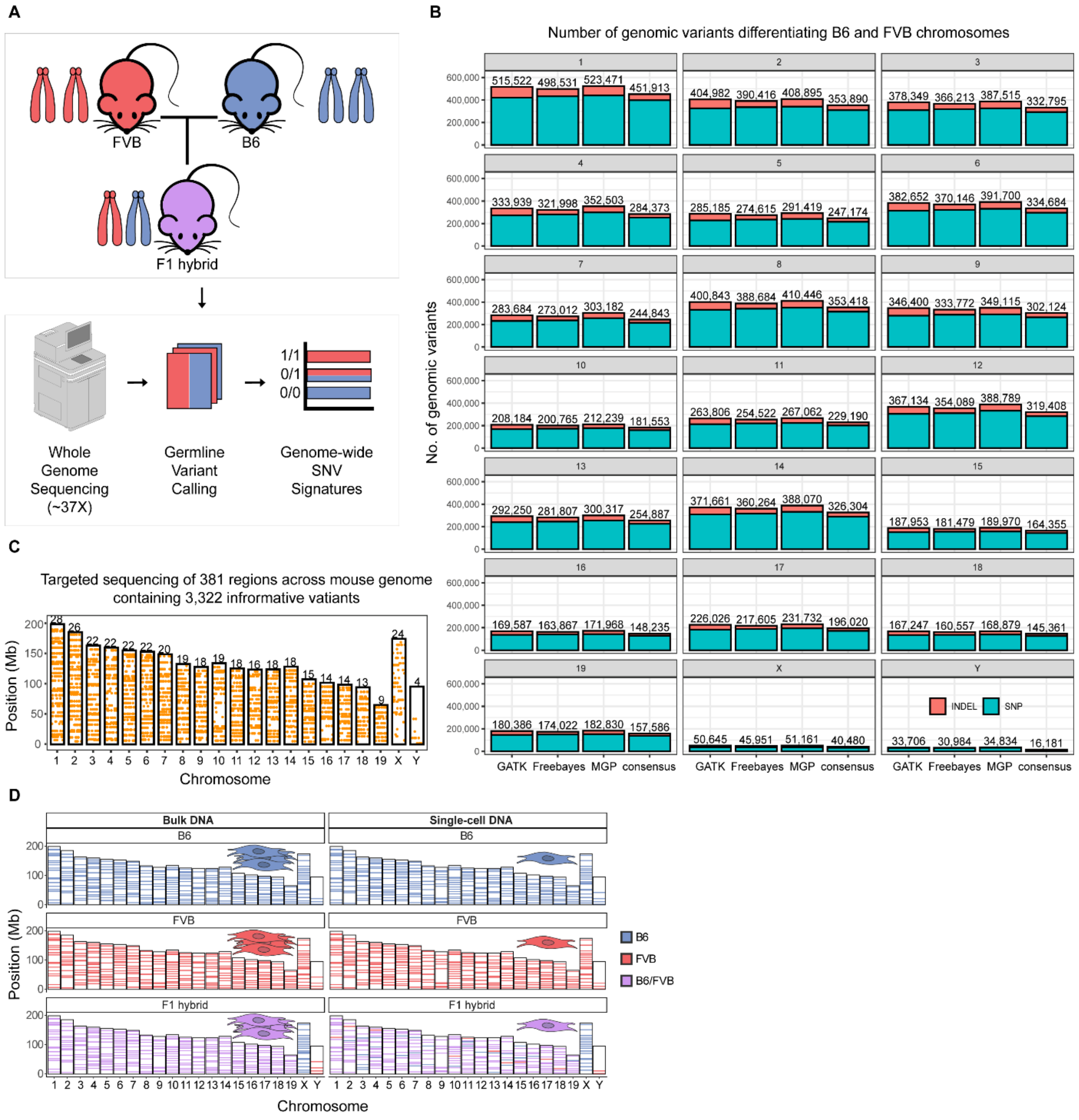

Fig. S1. Developing single-cell DNA sequencing assay for individual chromosome analysis. (**A**) Experimental design for developing individual chromosome identification by sequencing for this study. (**B**) Genome-wide consensus of short genomic variants used as an input for custom AmpliSeq sequencing panel design. Utilized germline variant calls from GATK and FreeBayes produced in this study, as well as the data set of germline variants from the Mouse Genome Project. (**C**) Location of targeted loci in the AmpliSeq sequencing assay designed for analysis of chromosome segregation patterns in our model of premature partitioning of diploid, non-replicated chromosomes. (**D**) Final Ampliseq panel readout from bulk and single-cell DNA samples. Lower right panel: the presence of both parental homologs (B6 and FVB) is evident

despite the fractional loss of heterozygosity due to allelic dropout during single-cell whole-genome amplification.

Table S1. (separate file)
